# The Anti-Tumor and Bortezomib-Sensitizing Effects of Apigenin in Multiple Myeloma

**DOI:** 10.3390/cimb47090717

**Published:** 2025-09-03

**Authors:** Ye Chen, Lan Wu, Siyu Wang, Huihao Chen, Miaojun Chen, Yanfen Huang, Bin Ding

**Affiliations:** School of Life Sciences, Zhejiang Chinese Medical University, Hangzhou 310053, China; cheny_0121@163.com (Y.C.); wulan001212@163.com (L.W.); wsyis541@163.com (S.W.); chh0041021@gmail.com (H.C.); wanzehua1@gmail.com (M.C.); hyf3797@126.com (Y.H.)

**Keywords:** apigenin, multiple myeloma, oxidative stress, apoptosis, bortezomib

## Abstract

Multiple myeloma (MM) is a kind of plasma cell neoplasm, accounting for approximately 10% of hematologic malignancies, with a high mortality rate. Apigenin (APG), a flavonoid, has been reported to have antiviral, antibacterial, antioxidant, and anticancer properties. However, the impact of APG on MM and bortezomib (BTZ) sensitization has not been investigated. The effects of APG on the proliferation, cell cycle, apoptosis, and oxidative stress of RPMI-8226 and U266 cells were investigated using CCK-8 assay, crystal violet staining, flow cytometry, Western blot, and PCR. It was observed that APG treatment increased the G1 phase cells, by which the expression of P21 increased, and the expression of CDK2 and Cyclin D1 decreased. Even though Necrostatin-1 (a potent necroptosis inhibitor) and Fer-1 (a ferroptosis inhibitor) could attenuate the effect of APG, the effect of Z-VAD-FMK (a pan-caspase inhibitor) was more significant. APG treatment increased the transcription of *P53* and *BAX*, and the level of cleaved-PARP1 and cleaved-Caspase 3 in two MM cell strains. In addition, the APG application could dose-dependently increase the ROS, MDA, and GSSH levels, and decrease the GSH level in both cell strains, by which the transcription of *GCLC*, *NQO1*, *GSTM2*, *NRF2*, and *GPX4* were attenuated. Finally, APG enhances the inhibitory effect of BTZ on MM cell growth. This study provides a potential therapeutic approach of APG on MM.

## 1. Introduction

Multiple myeloma (MM) is a heterogeneous B-cell malignancy characterized by clonal plasma cell proliferation in the bone marrow. Its complex biological mechanisms lead to profound immunosuppression, end organ damage, and high mortality [1,2]. Although immunomodulators, proteasome inhibitors, and anti-CD38 monoclonal antibodies have significantly improved survival, drug resistance and relapse remain major challenges [3]. Thus, discovering novel therapeutic agents and developing complementary strategies are urgent unmet needs in MM therapy.

Proteasome inhibitors (PIs) represent the most commonly used class of targeted therapeutics for MM [4]. The induction of oxidative stress has been recognized as a crucial mechanism underlying PI-induced apoptosis in MM cells [5]. Under oxidative stress conditions [6], excessive intracellular reactive oxygen species (ROS) levels can result in lipid peroxidation and damage of proteins and DNA [7]. The predominant mechanism, by which ROS exerts cytotoxic effects on tumor cells, involves the activation of programmed cell death (PCD), which is executed by a family of cysteine-dependent aspartate-directed proteases known as caspases. Through either extrinsic or intrinsic pathways, caspase-mediated PCD ultimately leads to the execution of apoptosis [8]. Cysteine protease 3 (Caspase 3) serves as the pivotal enzyme in apoptosis signaling cascades, and its activation constitutes a critical step in apoptotic signal generation [9]. During caspase-dependent apoptosis, poly (ADP-ribose) polymerase 1 (PARP1) undergoes cleavage by Caspase 3 into a DNA-binding fragment and a catalytic fragment. The DNA-binding fragment translocates into the nucleus, where it irreversibly binds to DNA, thereby inhibiting the repair function of PARP1. Simultaneously, the catalytic fragment, a cytoplasmic poly (ADP-ribose) (PAR) carrier, facilitates apoptosis-inducing factor (AIF)-mediated apoptosis [10]. Additionally, ROS can trigger apoptosis through inactivating or enhancing ubiquitination of the key anti-apoptotic protein B-cell lymphoma 2 (BCL2) [11].

**Figure 1:**
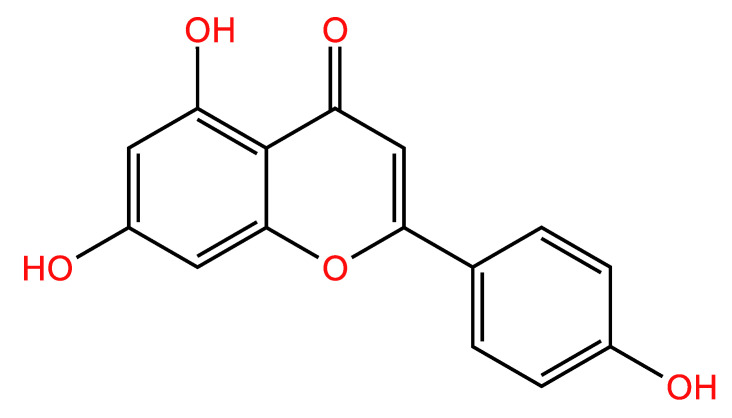
Chemical structure of apigenin (APG).

Apigenin (APG) (Figure 1), a natural flavonoid compound, exhibits multifaceted therapeutic potential through diverse biological mechanisms, including the induction of cell cycle arrest, promotion of apoptosis, anti-inflammatory effects, and antioxidant activity. APG induces cell cycle arrest at various proliferative phases (G1/S or G2/M) by modulating the expression of Cyclin-dependent kinases (CDKs) [12]. As established by previous research, APG regulates the intrinsic apoptotic pathway through Caspase 3 activation and subsequent initiation of programmed cell death [13]. Extensive studies have demonstrated APG’s antitumor efficacy in multiple cancer types, including bladder cancer [14], breast cancer [15], ovarian cancer [16], colon cancer [17], hepatocellular carcinoma [18], prostate cancer [19], and melanoma [20], by inhibiting proliferation of tumor cells and inducing apoptosis. However, the potential therapeutic application of APG in MM and its ability to sensitize cells to bortezomib (BTZ) remains unexplored. This study investigates the antitumor effects of APG against MM.

## 2. Materials and Methods

### 2.1. Reagents

APG was purchased from TargetMol Chemicals Inc. (Boston, MA, USA) and dissolved in DMSO to obtain gradually increased concentrations of 5, 10, 20, 40, 80 μM BTZ. chloroquine (CQ), necrostatin-1, ferrostatin-1 (Fer-1), and Z-VAD-FMK were purchased from MedChem Express (Monmouth Junction, NJ, USA).

### 2.2. Cell Culture

Human MM cell lines RPMI-8226 and U266, purchased from Meisen Chinese Tissue Culture Collections (Hangzhou, China), were cultured in RPMI 1640 medium (R8758, Sigma-Aldrich, Merck KGaA, Darmstadt, Germany) supplemented with 10% (*v*/*v*) fetal bovine serum (FBS, SA211.02, Biological Industrie, Beit Haemek, ISR) at 37 °C in a humidified incubator (Thermo Fisher Scientific, Marietta, GA, USA).

### 2.3. CCK-8 Assay

The Cell Counting Kit-8 (CCK-8, K1018, APExBIO, Shanghai, China) was used to assess the viability of MM cells. Briefly, 2 × 10^4^ cells were seeded in a 96-well plate.

APG Inhibits MM Cell Growth: Following treatment with various concentrations of APG for 24 h, 10 μL of CCK-8 solution was added to each well. The plate was then incubated at 37 °C for 20 min, and absorbance was measured at 450 nm using a microplate reader (Omega, model 415-2180, Deckenpfronn, Germany).

APG Potentiates the Anti-Tumor Effects of Bortezomib (BTZ): RPMI-8226 and U266 cells were treated with increasing concentrations of BTZ alone for 6 h. After BTZ removal, cells were either cultured for an additional 42 h or treated with 20 μM APG and cultured for a further 42 h. Subsequently, 10 μL of CCK-8 solution was added to each well. The plate was incubated at 37 °C for 20 min, and absorbance was measured at 450 nm using the microplate reader.

### 2.4. Crystal Violet Staining

Crystal violet staining solution (C0121, Beyotime, Shanghai, China) was also applied to assess the viability of MM cells. The MM cells’ treatment was identical to that described in 2.3. Briefly, the 20 μM and 40 μM APG-treated MM cells in a 96-wells plate were fixed with 4% paraformaldehyde for 10 min. After washing twice with distilled water for 2 min, the cells were stained with crystal violet solution for 15 min. After staining, the cells were washed with distilled water, and the cells were photographed under a microscope (SOPTOP, E31SPM, Ningbo, China).

### 2.5. Flow Cytometry for Cell Cycle Analysis

The Cell Cycle Assay Kit (C543, Dojindo, Shanghai, China) was used to determine the cell cycle. Briefly, MM cells were seeded in 6-well plates with a density of 5 × 10^6^ cells per well to grow overnight, and then treated with 40 μM APG for 24 h. Then, the cells were collected by centrifugation. After washing with phosphate-buffered saline (PBS), the cells were resuspended in 70% ethanol and fixed at 4 °C for 2 h. Again, the fixed cells were washed with PBS and centrifuged. The cells were finally resuspended with the working solution (500 μL Assay Buffer containing 25 μL PI Solution and 2.5 μL RNase Solution), and incubated at 37 °C and 4 °C in the dark for 30 min each, and then analyzed using a BD Accuri C6 flow cytometer (BD Biosciences, Franklin Lakes, NJ, USA).

### 2.6. Flow Cytometer Detection of Reactive Oxygen Species (ROS)

The ROS Detection Kit (S0033S, Beyotime, Shanghai, China) was used to assess the intracellular ROS levels. Briefly, MM cells were, respectively, treated with 20 μM and 40 μM APG for 24 h. After that, the cells were collected and resuspended with DCFH-DA solution. After incubation at 37 °C for 20 min, the cells were washed three times with serum-free culture medium and immediately analyzed with a BD Accuri C6 flow cytometer (BD Biosciences, Franklin Lakes, NJ, USA), and the ROS levels were determined using FlowJo software (version 10.0.7r2).

### 2.7. Determination of Apoptosis

Apoptotic cells were stained with annexin V-FITC/propyridine iodide (PI) and identified using an annexin V-FITC apoptosis detection kit (Beyotime, Shanghai, China). Then, 3 × 10^5^ MM cells per well were cultured in six-well plates and treated with corresponding concentrations of BTZ and APG. After 48 h, the cells were collected, washed with PBS, and suspended in 150 μL annexin V binding buffer. Then, they were fixed in 1.25 μL of annexin V-FITC and 1.25 μL of PI at 24 °C for 15 min. Apoptosis in each well was detected using FlowJo software (version 10.0.7r2).

### 2.8. Western Blot (WB) Analysis

Cells were lysed with RIPA lysis buffer containing phosphatase and protease inhibitors (AR0105-100, Boster, Wuhan, China). The lysates were centrifuged at 12,000 rpm for 15 min to collect the supernatants. The protein concentration of the supernatants was determined with the BCA Protein Assay Kit (P0009, Beyotime, Shanghai, China). The identity amount proteins of different cells assays were separated by 10% or 12% PAGE gels (PG112, PG113, Yanmei, Shanghai, China) and then transferred onto PVDF membranes (IPVH00010, Immobilon-P, Carrigtwohill, Ireland). After blocking with a protein-free blocking solution (AR0041, Boster, Wuhan, China) for 30 min, the membranes were sheared according to the molecular mass of the detected proteins and incubated with the specific primary antibodies overnight at 4 °C. In this study, the following antibodies have been engaged: anti-Cyclin-dependent kinase inhibitor 1A (P21) (1:1000, A19094, ABclonal, Wuhan, China), anti-Cyclin-dependent kinase 2 (CDK2) (1:1000, A18000, ABclonal), anti-Cyclin D1 (1:2000, ab40754, Abcam), Caspase 3 (1:3000, ab32351, Abcam), PARP1 (1:2500, ab191217, Abcam), anti-GAPDH (1:50,000, 60004-1-Ig, Proteintech), and β-actin (1:20,000, 4970s, Cell Signaling Technology). On the next day, the membranes were washed three times with TBS containing 0.05% Tween-20 (GC204002, Servicebio, Wuhan, China), and incubated with the corresponding secondary antibodies at room temperature for 1 h. After washing three times, E-ECL Oxidant (SQ203L-2, Yamei, Shanghai, China) and E-ECL (SQ203L-1, Yamei, Shanghai, China) Substrate solutions were pipetted onto the membranes for the chemiluminescence signal development.

### 2.9. Total RNA Preparation and RT-qPCR

The total RNA was extracted from MM cells with TRIzol reagent (Invitrogen, Carlsbad, CA, USA) and the concentration was determined with a Nanodrop (Thermo Scientific, Waltham, MA, USA). Subsequently, the mRNA was reverse transcribed into cDNA with ReverTra Ace^®^ qPCR RT Master Mix with gDNA Remover (TOYOBO, Osaka, Japan). The specific genes were amplified and real-time monitored by the StepOnePlus Real-Time PCR System (Applied Biosystems, Foster City, CA, USA) and relative transcriptional expression was calculated with the 2^−∆∆CT^ method. All primers applied in this study were synthesized by Sangon Biotech (Shanghai, China), and the primer sequences are listed in Table 1.

### 2.10. Determination of Oxidative Stress-Related Indicators

The RPMI-8226 and U266 cells were, respectively, treated with 20 μM or 40 μM APG for 24 h. The MDA and oxidized GSH/GSSG concentrations in cells were measured using the MDA or GSH/GSSG assay kits (Beyotime Biotechnology, Shanghai, China), respectively, following the manufacturer’s instructions.

## 3. Results

### 3.1. APG Inhibits MM Cell Growth

**Figure 2:**
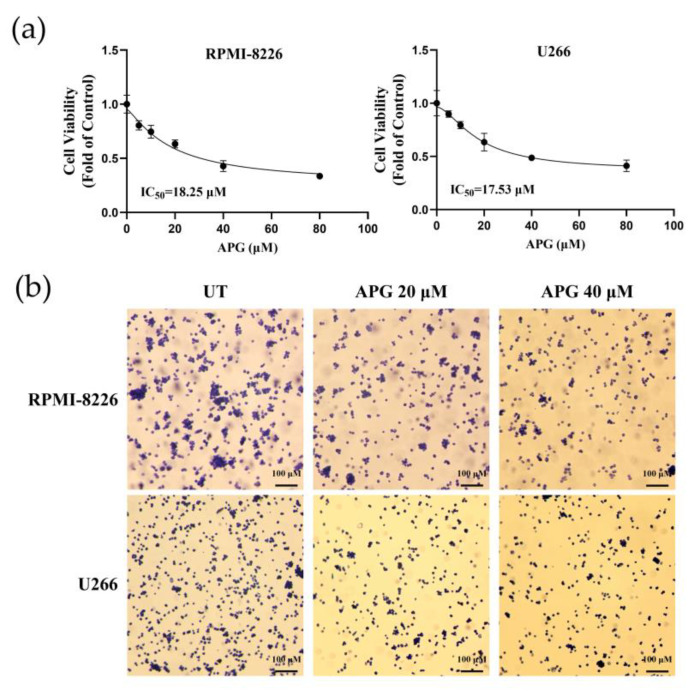
APG inhibited multiple myeloma (MM) growth: (**a**) CCK-8 assay showed that APG inhibited the proliferation of RPMI-8226 and U266 cells after continuous incubation for 24 h. (**b**) Crystal violet staining of MM cells treated with APG for 24 h. UT: untreated; APG: APG-treated.

Two MM cell lines, RPMI-8226 and U266, were used to evaluate the effect of APG on the proliferation of MM cells. In this work, RPMI-8226 and U266 cells were incubated with gradually increased concentrations of APG and cell viability was then measured using CCK-8 assays. The results of CCK-8 assays showed that APG inhibited the growth of MM cells in a dose-dependent manner. And the IC50 (50% inhibitory concentration) of APG on RPMI-8226 and U266 was 18.25 μM and 17.53 μM, respectively, which was calculated with the cell viability curve (Figure 2a). To further validate the anti-myeloma activity of APG, we performed crystal violet staining assays. Compared with the control group, treatment of 20 μM and 40 μM APG significantly reduced crystal violet-stained cells (Figure 2b), indicating that APG markedly inhibits the growth of MM cells.

### 3.2. APG Induces G0/G1 Cell Cycle Arrest of MM Cells

**Figure 3:**
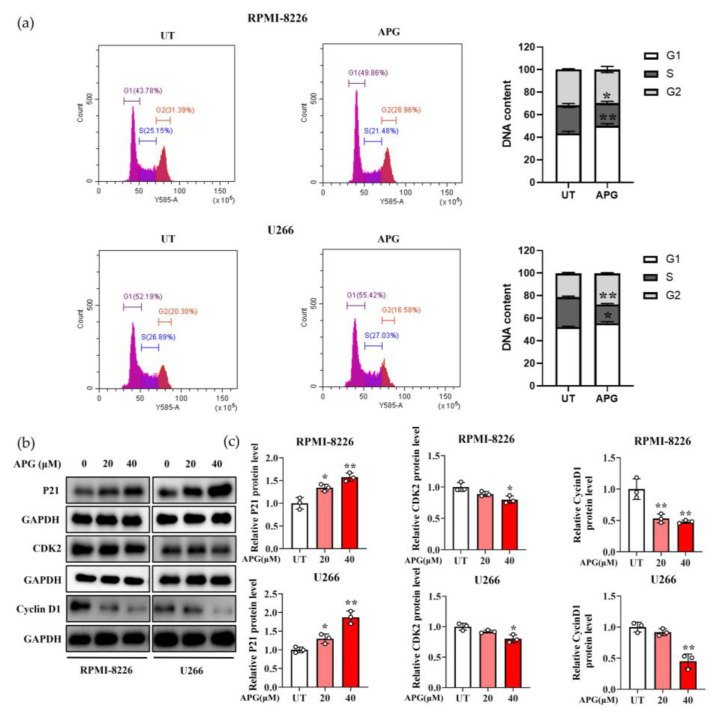
APG induces G0/G1 phase arrest in MM cells: (**a**) The APG treated MM cells, in the G1, S, and G2 phases, was detected by flow cytometry. (**b**,**c**) The levels of cell-cycle-regulating proteins were detected by Western blot (WB). All values are expressed as the mean ± SD of three independent replicates. * *p* < 0.05, ** *p* < 0.01.

To investigate whether the inhibitory effect of APG on MM cell proliferation was mediated through cell cycle arrest, we performed cell cycle analysis by flow cytometry. The results showed that APG treatment significantly increased the proportion of MM cells in G1 phase while decreasing the population in G2 phase (Figure 3a). We assessed the protein level of G1/S-Specific Cyclin D1 (Cyclin D1) and Cyclin-dependent kinase 2 (CDK2), two key regulators improving G1 to S phase transition (Figure 3b,c). Notably, APG treatment downregulated the expression of both Cyclin D1 and CDK2 and, on the contrary, stimulated Cyclin-dependent kinase inhibitor 1A (P21) expression. These findings indicated that APG suppressed MM cell proliferation by inducing G0/G1 phase arrest.

### 3.3. APG Induces Oxidative Stress in MM Cells

**Figure 4:**
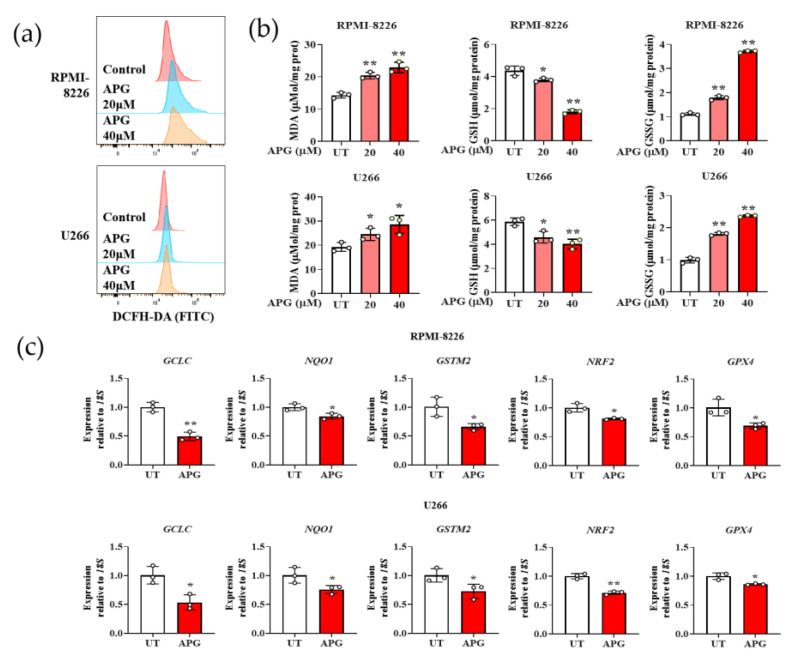
APG triggered oxidative stress in MM cells: (**a**) The effect of APG on ROS levels in MM cells was detected by DCFH-DA. (**b**) The effects of APG on the levels of MDA, GSH, and GSSG in MM cells. (**c**) The effects of APG on the transcription of *GCLC*, *NQO1*, *GSTM2*, *NRF2,* and *GPX4* in MM cells. All values are expressed as the mean ± SD of three independent replicates. *: compared with the untreated group. * *p* < 0.05, ** *p* < 0.01.

We evaluated the oxidative stress of APG treated MM cells with the DCFH-DA probe. The results showed that APG treatment stimulated ROS levels in both two types of MM cell strains in a dose-dependent manner (Figure 4a). Subsequently, we investigated the levels of Malondialdehyde (MDA), Glutathione (GSH), and Glutathione Disulfide (GSSG)—some of the hallmarks of cellular oxidative stress—in MM cells. The results indicated that the GSH content in APG-treated MM cells was significantly decreased. In contrast, the levels of MDA and GSSG were statistically increased (Figure 4b). In addition, we detected the transcriptional expression of proteins *GCLC*, *NQO1*, *GSTM2*, *NRF2*, and *GPX4* in APG-treated MM cells. The results showed that the transcription of these proteins in MM cells were reduced by APG treatment (Figure 4c). In summary, these results demonstrate that APG induces MM cells apoptosis by stimulating oxidative stress.

### 3.4. APG Promotes Apoptosis in MM Cells

**Figure 5:**
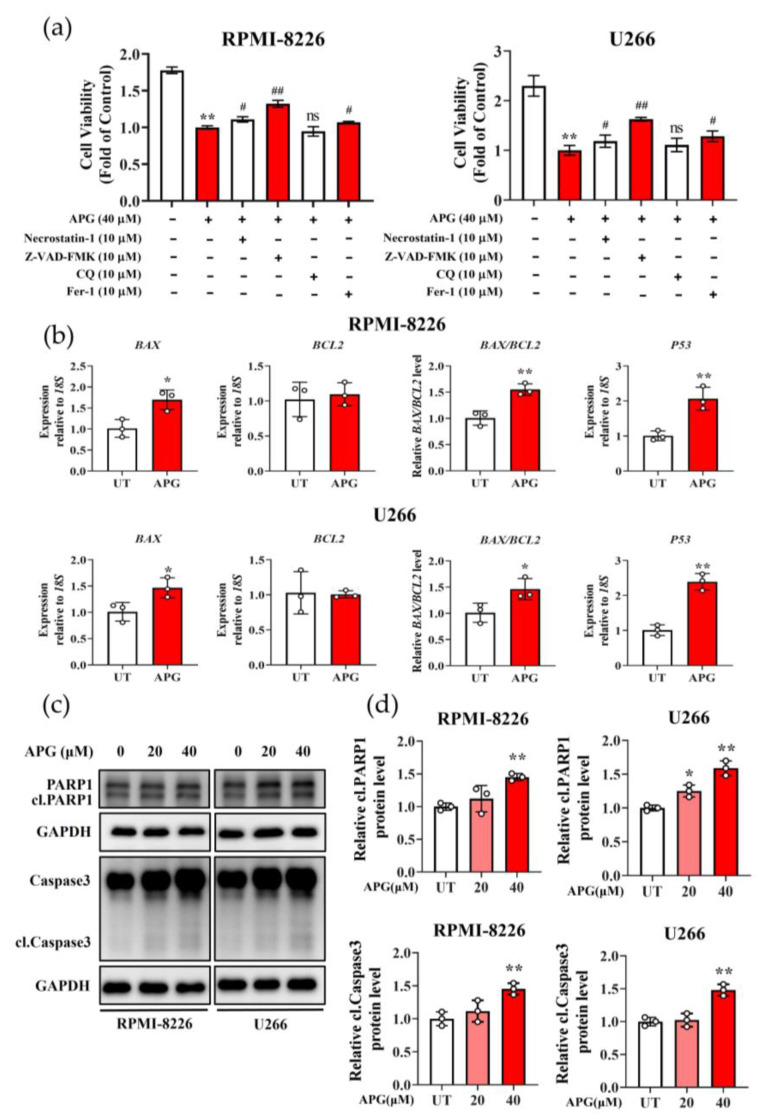
APG promotes apoptosis in MM cells: (**a**) The beneficial effects of Necrostatin-1, ZVAD-FMK, CQ, and Fer-1 on APG-induced cytotoxicity in MM cells. (**b**) The transcription of *BAX*, *BCL2*, and *P53* in APG-treated and untreated MM cells were determined by RT-qPCR. (**c**,**d**) The WB detection of the expression of PARP1, cleaved-PARP1 (cl. PARP1), Caspase 3, and cleaved-Caspase 3 (cl. Caspase 3) in APG-treated and untreated MM cells, which was quantified with ImageJ 1.54d. All values are expressed as the mean ± SD of three independent replicates. #: compared with the APG-treated group. *: compared with the untreated group. ns: not significant. * *p* < 0.05, ** *p* < 0.01, ^#^ *p* < 0.05, ^##^ *p* < 0.01.

We employed various inhibitors, Necrostatin-1 (a potent necroptosis inhibitor), ZVAD-FMK (a pan-caspase inhibitor), chloroquine (CQ, an autophagy inhibitor), and ferrostatin-1 (Fer-1, a ferroptosis inhibitor) to characterize the APG-induced cell death. Although Necrostatin-1 (cell recovery rate was 10.86%) and Fer-1 (cell recovery rate was 12.17%) could reduce the damaging effect of APG on MM cells viability, strikingly, ZVAD-FMK (cell recovery rate was 21.35%) showed the more pronounced protective effect against APG-induced cytotoxicity (Figure 5a). This indicated that APG predominantly triggers apoptotic cell death in MM cells. Since the ratio of pro-apoptotic protein BCL2-associated X (BAX) to anti-apoptotic protein BCL2 can indicate apoptosis, we detected the transcription of *BAX*, *BCL2*, and *P53* in both APG-treated MM cell strains with RT-qPCR. The results showed that following treatment with APG, *BAX* levels increased to 1.5-fold of control values, while *BCL2* expression showed no significant alteration; the *BAX/BCL2* ratio increased to 1.5-fold relative to controls. Separately, *P53* levels increased to 2.0-fold of control values, compared with the untreated cell assays (Figure 5b). Furthermore, the result of Western blot (WB) analysis demonstrated the significantly increased levels of the cleaved/activated forms of both Caspase 3 and PARP1 (Figure 5c,d). All these results suggested that APG could promote apoptosis in MM cells. But the underlying mechanism was still not discovered.

### 3.5. APG Potentiates the Anti-Tumor Effects of Bortezomib (BTZ)

**Figure 6:**
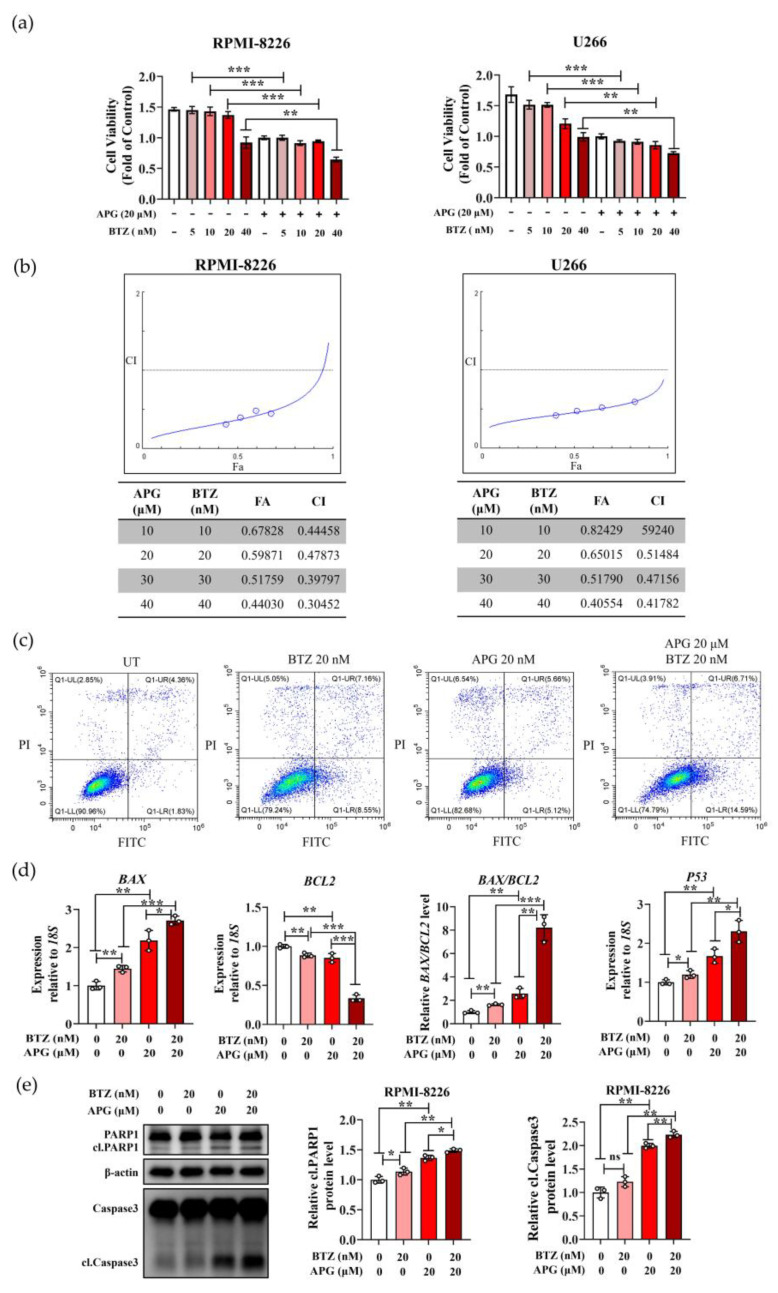
Apigenin increases multiple myeloma cell sensitivity to bortezomib: (**a**) CCK-8 assay demonstrating the viability of RPMI-8226 and U266 cells following treatment with BTZ alone or in combination with APG. (**b**) Combination treatments were performed in RPMI-8226 and U266 cells maintaining a constant ratio between the dose of the APG and BTZ, and cell viability was assessed at 48 h by CCK-8 assay. The combination index (CI) value and the relative fraction affected (FA) were determined at each dose combination (actual), and a simulation was run to estimate the CI value and confidence interval (—) across the entire FA range (simulation). CI < 1, CI = 1, and CI > 1 indicate synergistic, additive, and antagonistic effects, respectively. CI was calculated by the CompuSyn software program. (**c**) Apoptosis in RPMI-8226 cells was analyzed by flow cytometry. (**d**) The transcription of *BAX*, *BCL2*, and *P53* of RPMI-8226 cells following treatment with BTZ alone or in combination with APG were determined by RT-qPCR. (**e**) The WB detection of the expression of PARP1, cleaved-PARP1 (cl. PARP1), Caspase 3 and cleaved-Caspase 3 (cl. Caspase 3) in RPMI-8226 cells following treatment with BTZ alone or in combination with APG, which was quantified with ImageJ. All values are expressed as the mean ± SD of three independent replicates. ns: not significant. * *p* < 0.05, ** *p* < 0.01, *** *p* < 0.001.

RPMI-8226 and U266 cells were treated with increasing concentrations of BTZ alone for 6 h. After BTZ removal, cells were either cultured for 42 h or treated with 20 μM APG and cultured for 42 h. Cell viability was then determined using the CCK-8 assay. The results demonstrated that at equivalent BTZ concentrations, the combination treatment (BTZ followed by APG) significantly reduced the viability of MM cells compared to BTZ alone (Figure 6a). Next, we analyzed the synergism between APG and BTZ by applying CompuSyn (version 1.0). The combination of APG and BTZ showed synergistic anti-MM activity with a combination index (CI) < 1.0 (Figure 6b). Subsequently, apoptosis was analyzed by flow cytometry, and the results showed that compared with BTZ monotherapy, the combination treatment significantly enhanced cell apoptosis (Figure 6c). Additionally, transcriptional levels of apoptosis-associated genes *BAX*, *BCL2*, and *P53*, along with expression levels of apoptosis-related proteins Caspase 3 and PARP1 were examined in RPMI-8226 cells. The combination treatment significantly upregulated transcription of *BAX* and *P53*, downregulated *BCL2* transcription compared to BTZ monotherapy (Figure 6d), and increased cleavage of Caspase 3 and PARP1 (Figure 6e). These findings indicate that APG acts as a sensitizer, potentiating the anti-myeloma activity of BTZ.

## 4. Discussion

MM represents a clinically and genetically heterogeneous hematologic malignancy [21], exhibiting the high incidence and mortality rates among blood cancers, surpassed only by non-Hodgkin lymphoma [22]. Despite advances in proteasome inhibitors (such as BTZ) and chimeric antigen receptor (CAR) T-cell therapies, MM remains incurable due to drug resistance and toxicity [23,24,25]. It has been recognized that drug resistance is one of the major mortality factors in the clinic [22]. Notably, small molecules, such as flavonoids in herbs, have been increasingly applied in anticancer drug development and therapeutic applications due to their non-mutagenic properties in humans. Extensive studies have demonstrated that APG exhibits minimal toxicity to normal cells while displaying significant cytotoxicity against various cancer cell types. This selective anticancer activity enables the effective suppression of cancer stem cells in multiple malignancies [26]. Our results demonstrate that APG not only displays anti-MM activity but also can serve as a chemosensitizer for BTZ (Figure 6), effectively reducing drug resistance in MM.

The cell cycle represents a conserved mechanism for eukaryotic cell self-replication. Cyclin D/Cyclin-dependent kinase (Cdk) complexes play a pivotal role in cell cycle progression by coupling extracellular signals to the cell cycle machinery [27]. Cyclin D1 could complex with Cyclin-dependent kinase 4 (CDK4) and Cyclin-dependent kinase 6 (CDK6) to generate active kinase complexes which act early in the G1 phase, while Cyclin E is transcribed, translated, and complexes with CDK2 kinase, leading to progression through late G1 and entry into S phase [28]. This G1/S transition process is inhibited by P21 (the CDK inhibitor) through suppression of Cyclin E/CDK2 [29]. Our results demonstrate that APG treatment significantly downregulated Cyclin D1 and CDK2 expression while upregulating P21 in multiple myeloma (MM) cells (Figure 2b,c). This was concomitant with an increased proportion of cells in the G0 phase and decreased proportion in the S phase (Figure 2a), indicating that APG induces G0/G1 phase cell cycle arrest in MM cells, consistent with its reported effects in neuroblastoma and cervical cancer [30,31]. Notably, the cell cycle is closely associated with apoptotic processes, and several genes involved in cell cycle regulation, such as P53, also participate in apoptosis. The P53 tumor suppressor primarily functions during the G1 phase of the cell cycle, influencing cellular proliferation through its roles in cell cycle arrest and apoptosis [32]. Previous studies have demonstrated that APG induces P53 accumulation [33]. Activated P53 inhibits the G1-to-S phase transition by upregulating P21 expression. Additionally, it enhances the transcriptional level of BAX (a transcriptional target of P53) to promote apoptosis. As Schneider et al. reported [34], P53 can also modulate Cdk-activating kinase (CAK) activity, leading to CDK2 downregulation.

Excessive intracellular ROS generation frequently accompanies malignant transformation, leading to oxidative stress that may exert cytotoxic effects if left uncontrolled. Due to oncogene activation and enhanced metabolic activity, tumor cells typically exhibit elevated ROS levels compared to normal cells, rendering them more susceptible to chemotherapy-induced oxidative stress. An excessive ROS production leads to disruption of intracellular redox homeostasis, which can induce cellular apoptosis [35]. Our data show that APG increases intracellular ROS and MDA while reducing GSH (a key antioxidant) and increasing GSSG (oxidized GSH) in MM cells (Figure 4a,b). Concomitantly, APG downregulates the transcription of antioxidant proteins, including nuclear factor erythroid 2-related factor 2 (NRF2, a master regulator of redox homeostasis [36]), Glutathione peroxidase 4 (GPX4, an essential role in eliminating lipid peroxidation and exerting antioxidant effects [37]), and GCLC, NQO1, and GSTM2 (enzymes involved in GSH synthesis and detoxification) (Figure 4c). These findings demonstrate that APG can effectively induce oxidative stress in MM cells. The excessive accumulation of oxidative stress subsequently triggers apoptosis, thereby providing a promising therapeutic strategy for MM management.

The apoptotic program requires precise activation of multiple signaling cascades that are commonly disrupted in malignant cells. As such, pharmacological induction of apoptosis constitutes the predominant therapeutic mechanism for most clinically employed antitumor agents. Apoptosis, the primary mode of APG-induced cell death in MM, is supported by our inhibitor studies (Figure 5a). Molecularly, APG upregulates the pro-apoptotic protein BAX and downregulates the anti-apoptotic BCL2, increasing the BAX/BCL2 ratio (Figure 5b). This imbalance precipitates mitochondrial outer membrane permeabilization (MOMP), leading to the release of cytochrome c and subsequent activation of caspase cascades that execute the apoptotic program [38]. Poly (ADP-ribose) polymerase1 (PARP1), a nuclear enzyme, undergoes rapid activation in response to genotoxic stressors, including reactive oxygen species (ROS)-induced DNA damage [39]. During caspase-dependent apoptosis, PARP1 undergoes proteolytic cleavage, which simultaneously abrogates its DNA repair function and amplifies the apoptotic signal through multiple downstream effectors [40,41]. In our experimental results, the increased cleavage of Caspase 3 and PARP1, the key effectors of apoptotic execution, is consistent with the above. These results indicate that APG-induced oxidative stress activates apoptosis in MM cells.

Although APG has been shown to induce ferroptosis through mitochondrial pathways [42], trigger necrosis via mitochondrial dysfunction, ATP depletion, and elevated mitochondrial ROS production, and mediate autophagic cell death in human papillary thyroid carcinoma BCPAP cells [43], our findings indicate that these mechanisms are not predominant in MM (Figure 5a). Interestingly, APG-induced autophagy appears to exert a cytoprotective effect against apoptosis. Autophagy inhibition enhanced apoptotic cell death, thereby potentiating antitumor activity [44]. These findings suggest that a combination therapy strategy employing APG with autophagy inhibitors may represent a novel and effective approach for cancer treatment.

**Figure 7:**
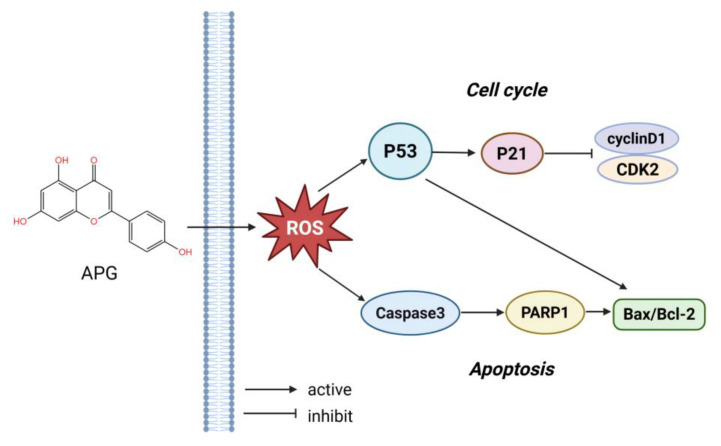
APG exerts anti-myeloma effects by triggering oxidative stress, inducing cell cycle arrest and apoptosis in MM cells.

In short, our study demonstrates that APG exerts anti-myeloma effects through multiple mechanisms, including proliferation suppression, cell cycle arrest induction, oxidative stress generation, and apoptosis promotion, while simultaneously potentiating the therapeutic efficacy of BTZ in MM (Figure 7). However, several limitations should be acknowledged. First, the precise molecular mechanisms underlying APG-induced apoptosis in MM cells remain to be fully elucidated. Second, the absence of in vivo validation through animal models represents a significant limitation of our current findings. Finally, clinical data are lacking to substantiate the potential therapeutic efficacy of APG in MM patients. These limitations highlight important directions for future research to further validate and expand upon our findings.

## 5. Conclusions

In this study, we investigated the therapeutic potential of APG in MM. APG treatment significantly inhibited proliferation, induced G1/S phase cell cycle arrest, triggered oxidative stress, promoted apoptosis, and enhanced BTZ sensitivity in MM cell lines. Notably, we provide the first experimental evidence demonstrating APG’s ability to effectively induce apoptosis in MM cells, underscoring its potential as a novel therapeutic agent for MM. However, the clinical translatability of these findings requires further validation. Additional studies are necessary to comprehensively evaluate the anti-myeloma efficacy of APG and its potential clinical applications.

## Figures and Tables

**Table 1:** Primer sets for qPCR.

Gene Name	Accession Number	Amplicon Size	Sequence (5′-3′)
*BCL2*	NM_000657	89	Forward: GGTGGGGTCATGTGTGTGG Reverse: CGGTTCAGGTACTCAGTCATCC
*BAX*	NM_138763	155	Forward: CCCGAGAGGTCTTTTTCCGAG Reverse: CCAGCCCATGATGGTTCTGAT
*P53*	NM_001126118	125	Forward: CAGCACATGACGGAGGTTGT Reverse: TCATCCAAATACTCCACACGC
*GCLC*	NM_001197115	79	Forward: GGAGGAAACCAAGCGCCAT Reverse: CTTGACGGCGTGGTAGATGT
*NQO1*	NM_001025433	196	Forward: GAAGAGCACTGATCGTACTGGC Reverse: GGATACTGAAAGTTCGCAGGG
*GSTM2*	NM_000848	99	Forward: TGTGCGGGGAATCAGAAAAGG Reverse: CTGGGTCATAGCAGAGTTTGG
*NRF2*	NM_001145412	174	Forward: TCAGCGACGGAAAGAGTATGA Reverse: CCACTGGTTTCTGACTGGATGT
*GPX4*	NM_001039847	100	Forward: GAGGCAAGACCGAAGTAAACTAC Reverse: CCGAACTGGTTACACGGGAA
*RNA18SN5*	NR_003286	158	Forward: ACCCGTTGAACCCCATTCGTGA Reverse: GCCICACAAACCACCAATCGG

Abbreviation: *BCL2*, B-cell lymphoma 2; *BAX*, BCL2-associated X; *P53*, Tumor Protein p53; *GCLC*, Glutamate-Cysteine Ligase Catalytic Subunit; *NQO1*, NAD(P)H Quinone Dehydrogenase 1; *GSTM2*, Glutathione S-Transferase Mu 2; *NRF2*, nuclear factor erythroid 2-relatedfactor 2; *GPX4*, Glutathione peroxidase 4; *RNA18SN5*, RNA, 18S Ribosomal N5.

## Data Availability

Data are contained within the article.

## Abbreviations

The following abbreviations are used in this manuscript:

AIF	Apoptosis-inducing factor
APG	Apigenin
BAX	BCL2-associated X
BCL2	B-cell lymphoma 2
BTZ	Bortezomib
CAK	Cdk-activating kinase
Caspase 3	Cysteine protease 3
CCK-8	Cellcountingkit-8
CDK2	Cyclin-dependent kinase 2
CDK4	Cyclin-dependent kinase 4
CDK6	Cyclin-dependent kinase 6
CDKs	Cyclin-dependent kinases
CQ	Chloroquine
CyclinD1	G1/S-Specific Cyclin-D1
GAPDH	Glyceraldehyde-3-Phosphate Dehydrogenase
GCLC	Glutamate-Cysteine Ligase Catalytic Subunit
GPX4	Glutathione peroxidase 4
GSH	Glutathione
GSSG	Glutathione Disulfide
GSTM2	Glutathione S-Transferase Mu 2
MDA	Malondialdehyde
MM	Multiple myeloma
NQO1	NAD(P)H Quinone Dehydrogenase 1
NRF2	Nuclear factor erythroid 2-related factor 2
P21	Cyclin-dependent kinase inhibitor 1A
P53	Tumor Protein p53
PARP1	Poly (ADP-ribose) polymerase 1
PCD	Programmed cell death
PI	Proteasome inhibitor
RNA18SN5	RNA, 18S ribosomal N5
ROS	Reactive oxygen species
WB	Western blot
